# Retraction Note: *TaWRKY40* transcription factor positively regulate the expression of *TaGAPC1* to enhance drought tolerance

**DOI:** 10.1186/s12864-023-09289-2

**Published:** 2023-04-06

**Authors:** Lin Zhang, Zhiyong Xu, Haikun Ji, Ye Zhou, Shushen Yang

**Affiliations:** College of Life Sciences, Northwest A&F University, Yangling, 712100 Shaanxi China

**Retraction Note**: ***BMC Genomics*20, 795 (2019).**10.1186/s12864-019-6178-z

The Editor has retracted this article. Concerns were raised regarding a number of figures, specifically:

- The WT specimen depicted in Fig. 4A appears to overlap with the Col-0 specimen under drought conditions shown in Fig. 4C in *Plant, Cell & Environment* [1].
- The TaGAPC1P-3/TaWRKY40 panel in Fig. 6C appears to overlap with the TaGAPCp3P-3/TaMyb panel of Fig. 9B in the *BMC Plant Biology* paper [2].
- Figure 2A (Col-0; drought 25d and 7d after re-watering) appears to overlap with Fig. 5A (WT; drought 25d and 7d after re-watering) of their *BMC Plant Biology* paper [2].
- Figure 2A (OE; drought 25d) in their *BMC Genomics* paper appears to overlap with Fig. 5A (OE-2 and OE-3; drought 25d) of their *BMC Plant Biology* paper [2].

The Editor therefore no longer has confidence in the results and conclusions of this article.

The authors have not responded to correspondence regarding this retraction.

[1] Zhang, L, Lei, D, Deng, X, Li, F, Ji, H, Yang, S. Retracted: Cytosolic glyceraldehyde-3-phosphate dehydrogenase 2/5/6 increase drought tolerance via stomatal movement and reactive oxygen species scavenging in wheat. *Plant Cell Environ*. 2020; 43: 836– 853. 10.1111/pce.13710.

[2] Zhang, L., Song, Z., Li, F. et al. RETRACTED ARTICLE: The specific MYB binding sites bound by *Ta_MYB in the _GAPCp2/3* promoters are involved in the drought stress response in wheat. *BMC Plant Biol***19**, 366 (2019). 10.1186/s12870-019-1948-y.

